# Dairy Goat Production Systems: A Comprehensive Analysis to Reframe Their Global Diversity

**DOI:** 10.3390/ani14243717

**Published:** 2024-12-23

**Authors:** Cesar A. Meza-Herrera, Cayetano Navarrete-Molina, Ulises Macias-Cruz, Gerardo Arellano-Rodriguez, Angeles De Santiago-Miramontes, Maria A. Sariñana-Navarrete, Ruben I. Marin-Tinoco, Carlos C. Perez-Marin

**Affiliations:** 1 Regional Universitary Unit on Arid Lands, Chapingo Autonomous University, Bermejillo 35230, Mexico; 2 Department of Chemical and Environmental Technology, Technological University of Rodeo, Rodeo 35760, Mexico; navarretemolina1977@gmail.com (C.N.-M.);; 3 Agricultural Sciences Institute, Baja California Autonomous University, Mexicali 21705, Mexico; 4 Department of Animal Production, Antonio Narro Agrarian Autonomous University, Laguna Unit, Torreon 27054, Mexico; 5 Rural Hospital No.162, Mexican Social Security Institute, Rodeo 35760, Mexico; 6 Department of Animal Medicine and Surgery, Faculty of Veterinary Medicine, University of Cordoba, 14014 Cordoba, Spain

**Keywords:** goat milk, adaptation, climate change, arid and semi-arid zones, smallholder

## Abstract

This review aims to emphasize the important role that goats and dairy goats play for many small-scale rural families worldwide, as well as to introduce a proposal for categorizing the main dairy goat production systems (DGPSs), using a multifactorial approach but emphasizing rainfall and nutritional supplementation level, as the focal categorization factors. The main DGPSs were divided into two metasystems based on available resources, each consisting of three production subsystems. In the first metasystem, the three subsystems have limited water, biotic, and economic resources, whose main economic rationality is based on reducing risk rather than maximizing outputs. In contrast, the three subsystems of the second metasystem usually have increased biotic, economic, and water resources, whose main emphasis involves maximizing product yield rather than reducing risk. The first metasystem involves DGPSs with a very limited or null nutritional supplementation: (a) subsistence, (b) extensive, and (c) agro-silvopastoral. The second metasystem includes those DGPSs with different levels of nutritional supplementation: (d) semi-extensive, (e) semi-intensive, and (f) intensive. There are numerous significant global initiatives focused on scientific collaboration and sharing information regarding nutrition, reproductive, and genetic technologies related to the safety and nutraceutical quality of goat milk and products while contextualized in different DGPSs. Hence, such scenarios should create additional opportunities for researchers, producers, policymakers, and development workers to come together and align interests and needs and exchange knowledge on effective goat farmer support strategies, environmental management, and consumer education. Undoubtedly, it is essential to reevaluate the DGPSs in the world since millions of producers and their families—most of them poor and marginalized—need this species, society needs their products, most of the worldwide arid and semi-arid lands need their recovery, and all of us should encourage the fulfillment of the sustainable development goals.

## Simple Summary

In arid and semi-arid regions of the world, goats are typically raised in rangeland conditions at latitudes above 30° N, dealing with significant annual fluctuations in the rainfall pattern, dictating, in turn, the quantity and quality of food availability, particularly in marginal goat production systems (GPSs). Under such a scenario, providing commercial grains and concentrates can be costly, making it difficult for most goat farmers to afford. Hence, the effective use of the rangeland biotic resources aligned with the amount of rainfall is key in determining the productivity of any production system, especially those with a dairy emphasis. We identified a compelling research opportunity focused on elucidating the key features that distinguish various dairy GPSs (i.e., DGPSs). Interestingly, DGPSs can be approached from the most traditional extensive system, resembling a more circular, less linear, more closed loop, mainly based on a circular economy approach and generally observed in developing economies, moving up to those less classical, highly industrialized, large-scale, linear-based dairy goat systems, mainly observed in developed economies.

## 1. Introduction

Globally, livestock production, in addition to generating about 40% of agricultural gross domestic product, contributes 33% of proteins to the human diet and 17% of calories consumed and generates important employment opportunities in rural households [1,2,3]. From a nutritional angle, in 2021, milk represented the second food of animal origin that contributed the most to global food security (FS), providing 39% of energy (kcal capita^−1^), 36% of protein (g capita^−1^), and 27% of fat (g capita^−1^) daily. Notably, in that year, milk contributed 96.3% of daily carbohydrates (g capita^−1^) [4]. Milk is considered one of the most complete foods, as it provides essential nutrients such as carbohydrates, proteins, fats, vitamins, and minerals. Additionally, milk plays a central role in infants, breastfeeding women, children, and the elderly, due to its easy digestion and absorption [5], making it one of the most valuable foods of regular consumption globally [6]. In 2022, bovine milk accounted for 81% of global milk production (MP), while dairy goat production (DGP) only contributed 2% [4]. However, DGP plays a crucial role in diverse regions, not only because of its nutritional value but also because of its cultural significance. Furthermore, goats play a significant role in the history of domestication, as they were among the initial species to form a beneficial connection with humans [7,8,9,10,11]. In recent years, DGP has grown rapidly, with a potential increase of 53% projected by 2030 [12], undoubtedly due to its well-recognized nutritional value [13,14,15,16]. Worldwide, DGP is typically categorized into three major production systems (i.e., extensive, semi-intensive, and intensive); still, each system presents unique management techniques and challenges [17,18,19,20,21]. Extensive systems, common in arid and semi-arid regions, rely on grazing, require minimal input, and face challenges such as limited access to water and poor pasture quality [17,19,22,23,24,25,26,27,28,29,30], as well as a high risk of inbreeding due to small herd size and uncontrolled breeding [31,32,33]. Semi-intensive systems, which combine grazing with some supplementary feeding, are typical in transition economies [24,25,26,27,34,35], while intensive systems, common in Europe and North America, involve confinement and advanced feeding methods, maximizing production efficiency using specialized dairy breeds [12,26,36].

Therefore, the understanding of dairy goat production systems (DGPSs) is essential to quantify the economic and sociocultural importance of the goat sector. However, DGPSs vary widely according to geographical, climatic, and socioeconomic conditions [37,38,39,40]. A key challenge in all DGPSs is the management of environmental impacts, in particular greenhouse gas emissions (GHGEs), which, despite being smaller in goats compared to large ruminants, require adequate and timely attention [41,42,43,44,45,46]. This scenario is aggravated by the environmental uncertainty generated by climate change (CC), especially in regions where DGP is essential for FS [7,12,23,26,47,48,49,50,51,52,53,54,55,56]. Therefore, the natural ability of dairy goats (DGs) to thrive in adverse conditions places them as an integral element when defining sustainable livestock production strategies [13,44,45,46]. Indeed, it is central to understand the evolution of DGPSs that have thrived into efficient, effective, resilient animal production systems with a low environmental footprint [12,23,26,47,52,53,54,55,57]. Worldwide, numerous studies have examined the evolution of diverse DGPSs [12,15,23,25,47,52,53,54,55]. Certainly, more than a decade ago, a previous report by our research group addressed the status quo and the main perspectives and challenges of DGPSs in the world [25].

However, it is crucial to examine these systems from different perspectives to evaluate their impact from a more comprehensive viewpoint to progress toward achieving sustainable development goals (SDGs). We aim to highlight the importance of the annual rainfall pattern in classifying global DGPSs as a key production strategy to tackle the SDGs, providing substantial economic and sociocultural benefits. Additionally, the important connection between rainfall patterns and DGPS performance will be highlighted to aid in decision-making, prioritize research funding, and create strategies for addressing changes in yearly rainfall trends. These issues undeniably affect millions of small-scale producers and their families [23,26,48,49,50,51,52,53,54,55]. This review aims to present a comprehensive analysis to reframe the global diversity of DGPSs and highlight the key role that dairy goats represent as a reliable, sustainable, and flexible option to guarantee real food security and contribute to the achievement of the SDGs in the 2030 agenda. Unless otherwise indicated, this research analyzes information generated from 2008 to 2022t by the FAO [4].

## 2. Dairy Goat Production: Distribution and Trends

### 2.1. The Worldwide Dairy Goat Inventory

**Figure 1:**
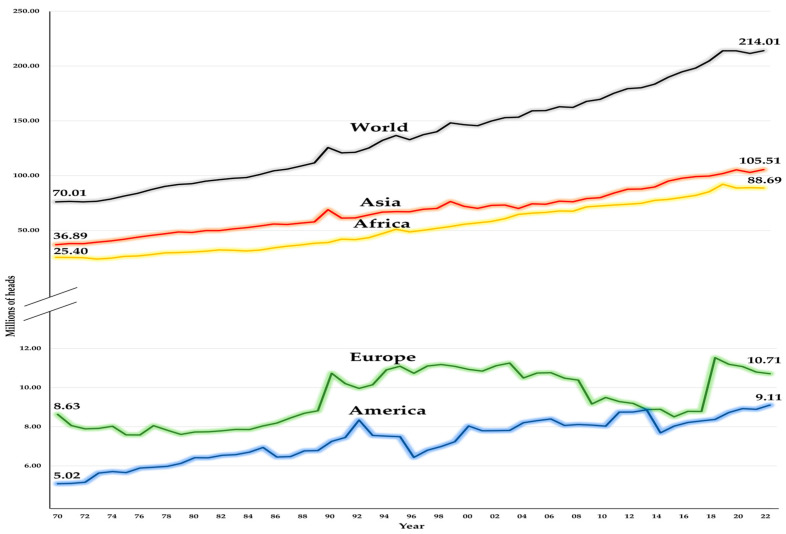
The global trend of the dairy goat inventory across continents from 1970 to 2022, millions of heads. Historically, the contribution of Oceania has been negligible, going from 400 heads in 1970 to 1357 heads in 2022.

According to FAO 2022, there were 1145.5 million goat heads (Mh) worldwide; from this amount, 214.01 Mh were classified as DGs. Analyzing the distribution and trends of the global DG inventory (GDGI), during the period 1970–2022, an increase in the number of goat heads was observed; it went from 76.07 Mh in 1970 to 214.01 Mh in 2022 (Figure 1) [4]. Historically, Asia has dominated the GDGI, representing an average of 50%, during the period analyzed (Figure 1). The African continent has the world’s second-largest inventory of DGs, with an annual average of 37%. During the period analyzed, Asia and Africa on average accounted for 87% of the GDGI (Figure 1) [4]. Of the top 10 countries with the largest inventory of DGs in the world, only 7 maintained their preponderance and belong to Asia (i.e., 4 countries) and Africa (i.e., 3 countries). These seven countries that accounted for 56.61% of the GDGI were India (17.34%), Mali (11.41%), Sudan (9.50%), Bangladesh 7.33%), South Sudan (3.78%), Indonesia (3.74%), and Pakistan (3.53%) [4]. On the other hand, Europe, with Greece (2.18%), Spain (1.45%), and France (0.97%), and The Americas, with Brazil (2.61%), Mexico (0.42%), and Haiti (0.36%), were the ones that made the largest contribution to the GDGI in the period analyzed. Finally, Papua New Guinea was the only country in Oceania to report DGs, with 1366 heads on average during the period under analysis [4].

### 2.2. The Worldwide Milk Goat Production

**Figure 2:**
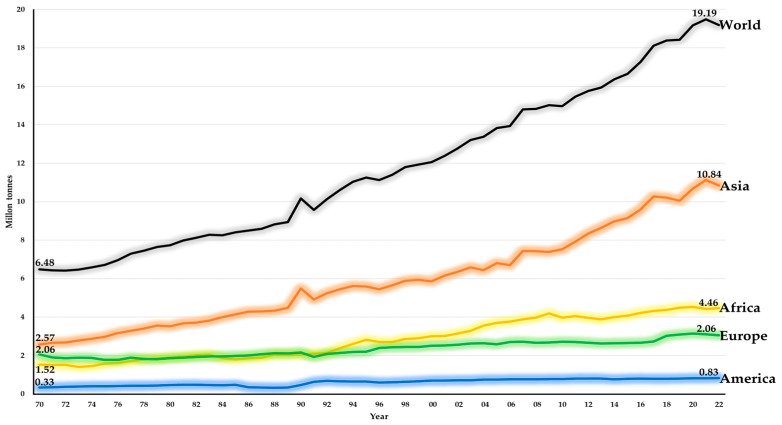
The global trend of dairy goat production across continents from 1970 to 2022, millions of tons. Historically, the contribution of Oceania has been negligible, going from 11 tons in 1970 to 39.89 tons in 2022.

Regarding the global production of goat’s milk (GM), it is important to clarify that, unlike bovine milk, GM does not have a deep-rooted culture of direct consumption. Certainly, GM is mainly used in the production of high-quality dairy products, which are generally intended for self-consumption or marketed as traditional products, sometimes with designation of origin [58,59]. According to FAO 2022, global GMP (GGMP) was 19.2 million tons (Mt), equivalent to 2.1% of global MP. Cattle and buffalo are undoubtedly the ones that have historically contributed the most to global MP, contributing 81.0% and 15.4%, respectively [4]. However, it is central to note that the GGMP experienced remarkable growth, close to 200%, over the last half-century, going from 6.5 Mt in 1970 to 19.2 Mt in 2022 (Figure 2). Additionally, even though the GDGI was 13.3% lower than that of dairy sheep, DGP was 52.6% higher than sheep’s milk [4]. Of the 19.2 Mt of GM produced in 2022, the first place worldwide was occupied by Asia (56.49%), followed by Africa (23.26%), Europe (15.93%), The Americas (4.31%), and Oceania (0.001%). Sustained growth of close to 29.5% was observed in the GGMP during the period under study, led by Asia (+45.93%), Europe (+14.74%), Africa (+12.39%), and The Americas (+9.47%). The only continent that showed a negative trend was Oceania (−5.02%), going from 42.00 to 39.89 t between 2008 and 2022, respectively (Figure 2) [4].

An interesting analysis of the GGMP considers the countries that make up the top 10 during the period under study. India contributed the most to the GGMP, with 32.11%, followed by Sudan (6.56%), Pakistan (5.00%), France (3.76%), Spain (2.93%), Bangladesh (2.84%), South Sudan (2.80%), Turkey (2.60%), Indonesia (2.09%), and the Netherlands (1.58%). On average, for the period analyzed, the top 10 accounted for 62.25% of the GGMP. Another interesting fact is that the top 5 GM-producing countries maintained the same hierarchy, with India, Sudan, Pakistan, Bangladesh, and France always at the top. Likewise, of the top 10, 5 countries are located in Asia, 3 in Europe, and 2 in Africa. The top 5 at the continental level are located in Europe, with Greece (2.47%) and Russia (1.46%), and in Africa, with Somalia (2.26%), Niger (1.92%), and Algeria (1.67%). Although no country in The Americas was listed among the top 10 of the GGMP, the main GM-producing countries were Brazil (1.57%), Jamaica (1.09%), Mexico (0.98%), Haiti (0.33%), and Bolivia (0.17%). Again, Oceania’s contribution to the GGMP was negligible [4].

### 2.3. The Worldwide Milk Goat Production Efficiency—Milk Produced Goat^−1^ Year^−1^

A more comprehensive analysis should consider not only the volume of milk produced by a country but also which continents and countries are more efficient in the production of GM, considering the volume produced per goat per year, between continents and within continents. FAO 2022 reported a global average yield per DG of 85.65 kg h^−1^ during the period under analysis; this value (i.e., kg h^−1^) was significantly exceeded by Europe (248.96), followed by much lower averages registered in Asia (82.58), The Americas (78.63), Africa (56.03), and Oceania (29.51). Undoubtedly, the above denotes a wide opportunity for improvement, considering what has been generated by Europe; indeed, with an inventory of only 5.01%, Europe contributed to 16.0% of the GGMP [4]. According to Pulina et al. (2018), this is due to the higher levels of innovation; commercialization; connectivity; systematization; and of course, a greater degree of specialization and scalability of the European goat industry [12]. In this same sense, during the last few decades, specialized breeds have been exported to many developing countries and have been crossed with local breeds, in order to improve MP [60]. Nonetheless, we need to mention that the GM yield values head^−1^ published by the FAO could present a certain degree of bias regarding the data reported by some countries. In fact, for instance, government statistics from Italy and Greece reported for 2013–2015 a GM yield of 159.69 and 260.42 kg h^−1^, respectively. This diverges from what was reported by the FAO for the same period in these countries, with corresponding values of 49.43 and 147.5 kg h^−1^ [4,61,62].

## 3. Dairy Goat Production: Main Production Systems in the World

Considering the definition of breed accepted by the FAO, Scherf (2000) reported the existence of 570 breeds of goats in the world [63]. However, Mason (1981) previously reported a total of 115 breeds, after eliminating the redundancy of breeds at the regional and global levels [64]. In this regard, in 2011, Devendra and Haenlein mentioned that, of the total breeds identified in the world, only 69 specialized exclusively in dairy production (DP) [65]. Additionally, they indicated that 36 (52%) were originally from Europe, 25 (37%) from Asia, and 8 (11%) from Africa. However, they clarified that most breeds have a dual specialization and that it generally involves the production of meat and milk [66]. Several research groups have reported that the valuable biological and productive characteristics of goats have led to an increase in interest in their production in both developing and developed economies, with the latter being where improved breeds with a clearly defined productive emphasis predominate, most often defined as “improved breeds” [49,66,67]. In general, at the global level, goats of Swiss, Spanish, French, and English origin have been considered the most valuable for DP from an MP perspective [68,69,70]. Some of the aspects that have slowed down the expansion of both the production and potential demand for GM have been associated with the perception that goat’s milk has a strong smell and taste, particularly when compared to cow’s milk (i.e., the “goat-like” taste and odor issue). Some studies have suggested that this perception goes back to the belief that GM is obtained in unhealthy conditions, and the byproducts are generated in deficient conditions and with low sanitary controls. Therefore, it is essential to generate education programs that highlight the benefits that GM brings to human health, a situation that could potentially change this negative perception that some societies have regarding GM and its derivatives; there is much to be done in this regard [66,71,72,73]. In addition, due to health problems related to human allergies to cow’s milk proteins, interest in GM has increased and contributed to the growth and development of DGs worldwide [26,58,66,71,73,74,75,76].

Considering the quantity and variability of GMP around the world, bibliographic research was carried out on the main scientific dissemination platforms, as well as in the institutional repositories of various universities located in the goat production belt (i.e., India, Bangladesh, Turkey, as well as Southern and Northern Mediterranean areas, Mali, Somalia, and Sudan). The above procedure was carried out to generate a holistic view of each of the DGPSs reported, as well as their main characteristics. Since diverse studies about goats mention that there are as many PSs as there are producers in the world, it is essential to systematize the main components that can define a PS. This information is essential to define the main strengths, threats, opportunities, weaknesses, and similarities in each of them, to promote an adequate integration that allows for the generation of a classification where these systems could be represented. Table 1 lists information from some of the DGPS classifications reported worldwide. Table 1 shows great variability and terminology used in the different classifications reported, making it difficult to achieve a categorization for DGPSs worldwide.

## 4. Dairy Goat Production Systems: Changing Paradigms, Using a Different Approach

Generally, the classification of animal production systems can be based on the intensity of the use of resources (i.e., extensive, intensive), the type of resource mainly used (i.e., pasture–forages or crop residues), the type of owner (i.e., social or private), their mobility scheme (i.e., nomadic or sedentary) or the type of product generated (i.e., milk, meat, dual purpose), among others. Globally, most DGPSs are extensive [25]. However, each PS is in a different agroecological region that receives different rainfall volumes and, consequently, diverse levels of nutrient availability [79,80,81]. Each PS is in specific regions whose markets demand specific products, mainly aligned with the two previous issues (i.e., rainfall volume, nutrient availability, and, therefore, level of supplementation). Consequently, the level of aggregation of the above roughly defines the objective or productive function of the PS; they modulate the product to be produced and offered [23,25,82]. As observed in Table 1, there are various classifications and typologies when trying to define a PS. With the large array of names in Table 1, we gained some insights from them to develop a new approach by combining different scenarios regarding both rainfall and biomass availability; the last, in turn, will allow us to determine whether a nutritional supplement should be provided or not. Undoubtedly, in terms of the environment, changes in climate—especially in rainfall patterns—were defined as key factors influencing the dynamics of socioecological systems due to the strong reliance on climate by land conditions and livelihoods [23,83].

As mentioned, we previously envisioned the importance of both rainfall volume and nutritional supplementation levels as central drivers when defining DGPSs [25]. Nonetheless, such initial ideas were not emphatically developed. Thereafter, the central role of both variables was certainly more evident as time went by, and the sustainability development goals (i.e., SDGs) were proposed. Therefore, an excellent opportunity emerged to strengthen this novel DGPS classification aligned with the SDGs. In particular, Goal 15 (Life on Land: protect, restore, and promote sustainable use of terrestrial ecosystems, sustainably manage forest, combat desertification, and halt and reverse land degradation and halt biodiversity loss) was foundational [84,85]. Deserts are among the “fragile ecosystems” addressed by Agenda 21, and “combating desertification and drought” is the subject of Chapter 12. Desertification includes land degradation in arid, semi-arid, and dry sub-humid areas, which results from various factors, including climatic variations and human activities [86]. Desertification affects as much as one-sixth of the world’s population, seventy percent of all drylands, and one-quarter of the total land area of the world [87,88]. It results in widespread poverty as well as in the degradation of billion hectares of rangeland and cropland [89,90,91,92]. The integrated planning and management of land resources is the subject of Chapter 10 of Agenda 21, which deals with the cross-sectoral aspects of decision-making for the sustainable use and development of natural resources, including the soils, minerals, water, and biota that land comprises [93,94]. This broad integrative view of land resources is essential for life-support systems as well as to enhance the productive capacity of the environment.

### 4.1. First Foundation: The Rainfall Level as the Cornerstone

As previously discussed, in arid and semi-arid areas, where the largest proportion of the world’s goat inventory is concentrated, rainfall is, most of the time, the only source of water available to trigger productive activities because its presence through rivers, lakes, or aquifers is not easily accessible [95,96]. Additionally, due to unpredictable climate variability, alterations in rainfall patterns are inevitable; while some areas encounter more frequent and intense droughts, others may see heightened rainfall frequency and strength [97,98]. In this respect, when evaluating different goat production scenarios, our research group has projected a reduction in worldwide rainfall by 2050, which will pose massive challenges in the global south, especially in arid and semi-arid areas. Hence, in the categorization put forth here, rainfall was taken as a compulsory component in defining or describing any specific production system. The hypothesis that supports this proposal is simple: rainfall defines the ecotype, and this, in turn—based mainly on its biotic and abiotic resources—defines whether a nutritional supplementation will be required to sustain the production of each of the proposed production subsystems.

### 4.2. Second Foundation: The Two Metasystems Approach

**Figure 3:**
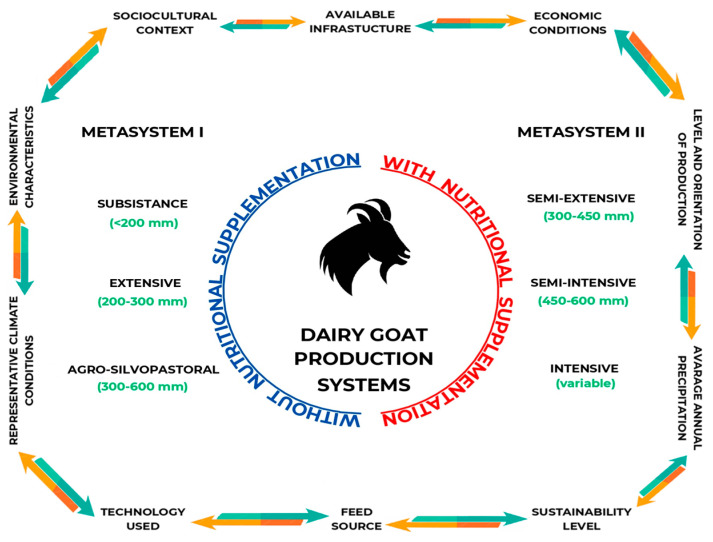
Rainfall volume and nutritional supplementation level as the main drivers in the proposal of the classification of the core dairy goat production systems in the world.

The previous information allows us to classify DGPSs into two large categories or metasystems: Metasystem 1—those that are developed in completely extensive environments, that is, that do not use any type of nutritional supplementation (i.e., forages and/or concentrates), and Metasystem 2—those that include a certain level of nutritional supplementation, i.e., that DGs may or may not be grazed for a period. This classification makes it possible to cluster the DGPSs according to their productive, technological, and sustainable characteristics, as well as to identify the regions where each system is predominant [23,24,25,26,27,34,35,77]. Moreover, a classification proposal that combines the previous issues will allow for the identification of these differences, the last being of paramount importance for the implementation of public policies, rural development programs, and market strategies adapted to the conditions and needs of each region [25,99]. Therefore, a DGPS categorization emphasizing the existence of a great diversity of farms, smallholder farmers, precipitation levels, ecotypes, and territories is presented in Figure 3. At some point, although this proposal can undoubtedly generate confusion, such diversity can be perceived more as an advantage rather than a disadvantage. Certainly, such variability can allow for a certain level of complementarity or connectivity between or among different subsystems and geographical areas, that is, an interesting opportunity to generate synergies between or among subsystems and ecotypes.

Another issue must be stated and clarified: the proposed classification should not be understood as a static approach; on the contrary, it must be understood as a dynamic entity subjected to changes and adjustments. The latter will allow for the generation of proposals for mechanisms that consent to the rapid and efficient monitoring of the various DGPSs; understand the changes they face; and propose solutions, adaptation, or mitigation measures [19,100,101,102]. Below is a description of each of the main DGPS proposed. As previously stated, they were divided into two metasystems depending on the resources available for each, with each metasystem consisting of three subsystems. In the first metasystem, the three subsystems were characterized according to limited water, biotic, and economic resources, with a shared focus on risk reduction instead of maximizing product output. In contrast, the three subsystems in the second metasystem typically have more access to water, biotic, and economic resources, leading to a focus on maximizing product yield instead of risk reduction in their economic rationale.

### 4.3. Metasystem I: Reduced Water Disposal (<200 up to 600 mm Annually) and Diminished Biotic and Economic Resources

The primary common factor within this goat production metasystem is the presence of water, biotic features, and economic scarcity in which its three subsystems (i.e., subsistence (transhumant), extensive, and agro-silvopastoral) operate [23,25,26,27,34,35]. Moreover, most of the time, they have an economic rationality that involves minimizing risk as another unifying component [103]. The goat plays a crucial role in these marginal production systems, as it efficiently utilizes the rangeland, which, due to its biotic traits such as overgrazing and abundant shrubbery, greatly benefits from the presence of goats from an ecological standpoint [7,8,9,10,11,25,26,27]. This is also crucial from a social and economic perspective, as goats enhance the potential for economic revenue for goat-keepers [23,25,26,48,49,50,51,52,53,54,55,78,81,103]. The annual water availability in this metasystem ranges from under 200 mm to 500 mm.

#### 4.3.1. Subsistence System or Transhumant (<200 mm)

This system, known as traditional, transhumant, or free-range, is most common in areas with less than 200 mm of average annual rainfall [23,24,25,35,76]. Transhumance is utilized to benefit from scattered and temporary forage resources, particularly in arid regions where it is prevalent [25,101,104,105]. Socioculturally, it is contextualized as the typical DG production (DGP) of rural and marginalized communities, where GP is essential for family survival on a day-to-day basis. Rooted in a foundation of self-reliance and mutual support, DGP is deeply linked to local culture and traditions [23,24,25,37,38,39,40,78,106,107]. It predominates in rural and marginalized regions of developing countries, where subsistence livestock farming is the norm. It generates extremely low income, with limited profitability that depends on the ability to cover the basic needs of the producing families [23,106,107]. As it is based on the use of natural resources (NRs), this system depends almost exclusively on these biotic resources [23,34,35,78]. This system produces low GHGE levels by not using external inputs or modern technologies. Recycled waste is commonly used in the local area to reduce environmental damage and encourage a circular economy [36,85,108]. There is a high level of biodiversity in the ecosystems in which it thrives, with methods that support the conservation of the natural environment and indigenous species. DGPSs focus mainly on preserving traditional knowledge and boosting biodiversity [25,30,33,56,57,78,102]. This subsistence system can be found in areas with extreme climates, where conditions are frequently difficult and changing [25,78]. It is characterized by great adaptability and resilience, with methods tailored to specific local circumstances [12,23,26,47,52,53,54,55,57]. Their food source includes natural resources like indigenous grasses and other types of plants. The system lacks infrastructure and technology, relying mainly on traditional, inherited, or ancestral practices for herd management [25,78]. The DP per head (DP h^−1^) is typically low, often used for family consumption, or sold in small, local markets. Dairy byproducts frequently have cultural and ceremonial significance. This system primarily utilizes local and criollo-creole breeds [23,24,25,37,38,39,40,58,106,107]. One of the advantages of this system is its strong commitment to environmental, cultural, and social sustainability, emphasizing the importance of maintaining traditions and lifestyles [60]. While it is found in numerous global regions, it is particularly prevalent in South Asia, Africa, and some parts of Latin America [23,25,44,46,56,76,102,105].

#### 4.3.2. Extensive System (200–300 mm)

This system is also referred to as a daytime grazing system with night confinement and no supplementation or an extensive system with minimal inputs [23,78]. It is common in areas with an average yearly precipitation of 200–300 mm, particularly in semi-arid or arid areas, where there is limited forage and a small number of animals. This setup is seen in undeveloped rural areas, with the economy centered around animal husbandry [23,24,25,37,38,39,40,58]. Communities heavily depend on general practitioners for their livelihoods, which are crucial for their cultural and social identity [25,78]. This system is prevalent in areas with underdeveloped agricultural economies, where farming and raising livestock are the primary means of income [25]. Similarly, it produces minimal revenue and is only profitable to a certain extent, relying on the presence of NRs and the weather conditions [23,25,35]. In places where this system develops, DGPSs serve as a means of survival rather than a major source of revenue. In the environmental setting, this is a heavily degraded production system from biotic, edaphic, and economic viewpoints, predominantly reliant on grazing vast expanses of natural terrain [17,19,22,23,24,25,26,27,28,29,30]. Their low GHGEs are a result of the provision of a pasture-based diet and traditional livestock management practices [36,85,108]. Waste is limited and typically blends seamlessly into the ecosystem, positioning it within a circular economy framework [108].

This system also promotes the preservation of indigenous species and the richness of the environment, leading to a significant biodiversity in this specific system despite its small scale and low input production [23]. In terms of weather and ability to adjust, this system is predominantly found in arid and semi-arid areas where rainfall is scarce and extreme climate conditions, including hot temperatures and significant seasonal climatic variations. This is why it displays a strong ability to withstand unfavorable weather conditions, using traditional techniques suited to the limited availability of water resources [25]. The NRs available through extensive grazing serve as the food source for this system. The existing infrastructure (INFRA) is limited, with DGs being exerted over vast expanses of grassland or grazing land, with minimal utilization of technology; this traditional system continues to be the most prevalent DGPS worldwide [23,24,25,78]. This mix of elements results in a low DP h^−1^ while also having low operational expenses. Dairy goats play a crucial role in the local diet of the inhabitants of these regions while holding cultural significance beyond just providing food [37,38,39,40]. Herds typically comprise local breeds, and they also might be characterized by limited connection to the markets. This system exhibits strong ecological sustainability, minimal environmental impact, and effective utilization of natural resources [23,25,35,78]. Sub-Saharan Africa, the Middle East, Central Asia, and parts of Latin America are the regions where this system is found [23,25,44,46,56,76,102,105].

#### 4.3.3. Agro-Silvopastoral System (300–600 mm)

This system is also referred to as a silvopastoral, pastoral, extensive agro-silvopastoral, or organic production system [23,24,25,36]. This kind of system is common in ecotypes where the average yearly precipitation ranges from 300 to 500 mm. The increased water availability enables producers to integrate agriculture, forestry, and livestock in regions where they combine DGP with forest and crop management [25,36]. Active participation and strong collaboration among community members are needed to ensure the sustainable management of resources [76]. Economic conditions in areas where it is implemented are typically rural regions in emerging and developing economies, with economic diversification playing a crucial role [23,24,25]. This system includes varied earnings from various sources (i.e., milk, timber, fruits, etc.), with a moderate level of profitability, although consistently maintained [25]. The sustainability and resilience of the system are associated with the economic advantages. Considering the environmental factor, this system efficiently utilizes NRs by emphasizing sustainable land use and incorporating trees and shrubs to enhance productivity [12,23,26,47,52,53,54,55,57]. The low GHGEs are attributed to the abundance of woody vegetation, which also enhances carbon sequestration capabilities. Waste is naturally recycled, enhancing soil productivity [108]. This system exhibits a high level of ecological biodiversity that supports various plant and animal species living together, thus enhancing the ecosystem’s ability to recover from disturbances, an augmented resilience [23,24,25,36,100]. The system promotes the integration of natural resource management with DGP to maximize environmental, biotic, and economic benefits through agroecological practices [23,24,25,34].

The agro-silvopastoral system thrives in tropical and subtropical climates that have defined rainy and dry seasons, providing an ideal environment for the sustainable growth of trees, grasses, and shrubs [25]. Furthermore, it is associated with a high ability to adjust to changes in climate by incorporating various layers of vegetation that shield the ground, retain water, and conserve moisture [23,24,25,36,100]. This system incorporates trees and shrubs into grazing areas, offering both shade and food for goats. The INFRA is simple but effectively used along with the management of NRs to enhance the utilization of land and/or territory [25]. Technology is not widely used, but some creative methods are employed in managing both the land and the herd [23,24,25]. This system prioritizes resilience and ecosystem conservation over income maximization, displaying a moderate degree of goat milk production [12,23,26,47,52,53,54,55,57]. Goat milk and its byproducts are included in a varied diet for self-consumption, while both milk and derivatives are highly appreciated for their nutritional value and for being based on clean, green, and ethical production schemes [77]. Typically, breeds like creole-criollo or indigenous genotypes are crossed with specialized breeds for specific markets, although the focus is on milk production. It prioritizes environmental sustainability by emphasizing soil conservation, ecosystem preservation, biodiversity protection, and supporting cultural and social values [37,38,39,40]. These DGPSs can be found primarily in Latin America, Africa, and certain areas of the Mediterranean region [21,23,25,34,44,46,56,76,102,105].

### 4.4. Metasystem II: Increased Water Disposal and Enlarged Biotic and Economic Resources

In general, in this metasystem, producers have capital as their main unifying element and possess an “economic rationality” focused on the maximization of the product obtained [23,24,25,27,35]. The three subsystems that constitute it, in different gradients, have economic and biotic resources that allow it to artificialize the goat production subsystems at different levels and exercise a certain control in the market of the products they offer [23,24,25]. They have land, irrigation water (either gravity or pumping), technological production packages with different levels of forage production, and particularly a greater economic capacity [23,24,25,36,54,77]. The water availability in this metasystem is from 350 to 600 mm, up to unrestricted water access.

#### 4.4.1. Semi-Extensive System (300–450 mm)

This system is developed in ecotypes that receive an annual rainfall between 350 and 450 mm; it is known for its more regulated management compared to extensive systems, with some occasional supplementation [34,35,77]. Referred to as a daytime grazing system with night confinement, this grazing approach emphasizes self-sufficiency and the local market, highlighting the crucial role of the local community and the strong connection between local producers and consumers [12,23,24,25,26,27,35]. Similarly, conventional customs are upheld, albeit with some adjustments to fit the economic conditions of the area [23,24,25,77]. This system can be found in developing economies, where a combination of livestock farming is popular [25,27]. Variable profitability is demonstrated based on the ability to reach markets and the consistency of climate conditions; it relies more on NRs than external inputs [12,23,24,25,34,35,77]. Its GHGEs are relatively low because of the emphasis on grazing and the limited use of concentrates and forages [108,109]. The generation of organic waste is reduced and spread out, making it easier to reintegrate into the ecological system [23,83,110]. Having a high environmental benefit, this system enables the coexistence of DGP with native plant and animal species [12,23,24,25,34,35]. Moreover, it demonstrates an emphasis on rotating grasslands and preserving ecosystems to uphold ecological equilibrium. This system exists in the Mediterranean and subtropical climates, where precipitation supports the growth of grasslands throughout most of the year [23,24,25]. It is a system that is highly adaptable to changes in the weather but relies on the quantity and pattern of rainfall [27]. The food supply involves grazing on native plants along with minimal additional feeding [12,23,24,25,34,35,77]. The INFRA and technology employed in this system are moderate, as they involve partial stabling with year-round access to pastures, demonstrating a focus on nutritional and reproductive herd management [23,34,35]. The milk production head^−1^ level in the system is moderate, focusing on achieving sustainability through balance; these DGPSs are important to improve local food security [23,24,25,27]. In this system, most goats are crossbred, showing an average sustainability level, mainly based on utilizing grasslands efficiently [34,35,77]. This system can be found in Latin America, particularly in Mexico and Brazil, as well as in certain Mediterranean areas [23,25,27,35,36,77].

#### 4.4.2. Semi-Intensive System (450–600 mm)

This system is connected to ecotypes that have annual precipitation ranges between 450 and 600 mm. It is commonly found in regions where there is a higher abundance of water and food sources [12,27]. The administration is more focused on technical aspects, such as pens, dietary supplements, and increased veterinary attention [25,35,36]. It is also known as a grazing system with forage supplementation, an intensive agro-silvopastoral system, an intensive grazing system, an intensive system with associated crops, a semi-intensive system with moderate inputs, and a semi-extensive system with high inputs [12,25,26,27,35,36]. In developing countries, this system is prevalent in the sociocultural context, with livestock farming being essential to the local economy [37,38,39,40]. Additionally, the local community engages in production actively, promoting cooperative networks among producers. This is why livestock plays a key role in the economy of regions with these DGPSs [36]. The system’s economic viability is moderate, with various sources of income, such as selling milk and its byproducts in nearby markets [12,36]. Efficiency in management and reducing costs are crucial for achieving success. This system utilizes NRs moderately along with external inputs to reduce strain on water and energy resources, resulting in moderate GHGEs and waste production [110,111,112]. This system enables some interaction with the natural surroundings, resulting in a moderate ecological balance. Yet, it showcases the adoption of sustainable methods for managing grasslands and utilizing intermediate technologies for environmental purposes [26,27,36]. It is found in temperate and Mediterranean regions, where the seasons allow for grazing and supplementing during tough times [23,24,25]. A specific level of climatic consistency is needed, including sufficient rainfall to maintain healthy pastures and adjust to seasonal changes. The primary sources of food consist of a mix of foraging in native plants and meadows supplemented with nutrients [23,34,35]. The INFRA is adjusted for partial stabling and access to pastures and agricultural areas for part of the year [23,25,34,35]. The system utilizes technology moderately for milking, in conjunction with managing the herd’s nutrition and reproduction [23,24,25]. The latter allows for a moderate level of milk production h^−1^, stressing an equilibrium with efficiency. They produce milk to create goods for local markets and sometimes distribute their products and byproducts outside their region [35,36]. The herds consist of mixed phenotypes with a strong presence of specialized breeds, demonstrating a significant presence in various markets such as local, regional, and superregional [23,27,77]. This system is primarily found in the Mediterranean area and other parts of the globe, particularly in Latin America [25]. However, it is becoming more popular in developed economies, as consumer needs call for the advancement of sustainable herd management techniques, along with the observed rise in the costs of the inputs required by these DGPSs [25,27].

#### 4.4.3. Intensive System with Variable Precipitation and Unrestricted Water Access

This system is developed under different average annual precipitation schemes and managed through confinement with regulated feeding and continual nutritional support. This DGPS is also known as confinement, intensive-industrial, and intensive system with or without associated crops [23,24,25,35,36,77]. It is typically found in irrigated regions, with moderate-to-high levels of hydrological technification, using surface water or water pumped from underground [23,26,35]. This system is most common in developed economies, where most of the DGPSs are owned by companies with substantial investments in technology and management, along with a strong focus on innovation, integration, and escalation [12,23,24,25,26,34,35,36]. Hence, the involvement of the residents in the nearby villages is restricted to being part of the workforce. Interestingly, however, this DGPS can also be found in certain developing economies, with a significant level of integration and access to worldwide markets [23,24,25]. Although they yield high profits per unit produced, they also require considerable financial investments, and their success hinges on their effectiveness and entry into advanced markets [25,35,36,77]. This DGPS needs increased water and energy usage because of its specific INFRA and highly intensified management [23,24,25]. There is a notable emission of GHGs, particularly methane and nitrous oxide, resulting from the use of concentrates and the management of manure. Furthermore, it generates a significant quantity of waste, such as manure and sewage, which needs appropriate handling to prevent pollution [109,110,111,112,113,114]. It is typically isolated from the surrounding environment; thus, the ecological biodiversity of this system is low. Due to the strict environmental regulations typically followed by advanced economies, this DGPS incorporates technologies focused on reducing emissions, treating waste, and improving energy efficiency [109,111]. Due to its specialized infrastructure, this system can operate in various climates. Feeding and livestock management are conducted in controlled and intensive environments, so it is not directly affected by external climate conditions [23,26]. The primary diet consists of high-quality concentrates, supplements, and forages, with the incorporation of state-of-the-art technologies for herd management, health, and reproduction control, all in conjunction with automated milking [23,24,25,26]. This results in a strong emphasis on production efficiency and milk quality, resulting in a high level of milk production h^−1^ [23,24,25,26,35,36,77]. This system yields high-quality products for local, national, and global markets, utilizing specialized breeds, and is fully integrated into supply food chains. However, the sustainability of the system may be compromised if any key components are disrupted within the production and market chain [23,25]. Nevertheless, it is important to note that the majority of these DGPSs have shown to be more environmentally friendly compared to other dairy production systems involving cows, sheep, or even water buffalo [45,115,116]. This DGPS is primarily situated in designated European nations, specific regions in the United States, and particular focal points in Latin America and Asia [23,24,25,26,27,35,36,77].

## 5. Conclusions

Diverse goat production systems, specifically those focused on milk production, are critical in supporting nutritional security for small farmers in diverse ecotypes, specifically in the arid and semi-arid regions around the world. These areas are mainly inhabited by small-scale producers who face high levels of marginalization and vulnerability. Goats overall, and specifically dairy goats (DGs), are known for their impressive ability to not only survive but also thrive, and even flourish, in harsh climatic and challenging environmental conditions, all while producing high-quality and nutritious products with minimal environmental footprint. Similarly, goat milk is well known globally for its rich nutritional content and easy digestibility, presenting great potential for sustainable growth in diverse rural areas around the world. So, DGs play a vital role in tackling food insecurity and encouraging sustainable development in these socially and biologically depressed areas. Thus, it is crucial to examine these systems from various angles to assess their effects from a broader perspective, aiming to enhance the sustainability of dairy goat production systems (i.e., DGPSs) and to fulfill the SDGs. This comprehensive review enhances our comprehension of the complex processes that dictate the arrangements of diverse biotic and abiotic resources that characterize the main DGPSs. Our commitment is to facilitate a complicated yet essential endeavor in categorizing global DGPSs as a crucial strategy to address the primary challenges that various DGPSs encounter. The latter is central to comprehending their continued ability to deliver significant economic and sociocultural benefits. This review particularly examines the crucial connection between the volume of rainfall received, the nutrient accessibility created in various ecotypes, and the level of nutritional support each DGPS requires to achieve its production objectives. These elements are essential in shaping every DGPS and play crucial roles in their growth, progress, health, and sustainability. Indeed, these problems indisputably impact millions of small-scale producers and their families.

Two main metasystems were proposed, primarily categorized according to the availability of water. The first metasystem and its three subsystems (i.e., subsistence (transhumant), extensive, and agro-silvopastoral) not only encounter limited water resources (i.e., less than 200 to 500 mm annually) but also struggle with limited biotic and economic resources. Therefore, their focus on an economic–productive rationale involves minimizing risk rather than maximizing productivity. These kinds of subsystems are defined by marginality as the primary common factor. Their degree of integration, organization, and scalability is almost non-existent or, at best, in its early stages. The limited, inadequate, and unreliable attempts involving these systems have been centered on seeking a say but not a vote in the regulation of the milk and meat markets. In contrast, the second metasystem and its three subsystems (i.e., semi-extensive, semi-intensive, and intensive) not only have more water availability (i.e., 350–600 mm, up to unlimited water access) but also have enlarged biotic and economic resources. Hence, their economic and productive rationale primarily focuses on maximizing productivity output. One key factor that bridges these subsystems is the capital accessible for investing. In the context of milk production, these subsystems show an increasing level of organization, integration, and scalability in product industrialization, market accessibility, and price of their products while maintaining complete control over the prices of sires and replacement females for sale.

The remarkable resilience of DGs in extreme weather conditions, paired with their genetic aptitude for efficient milk production, cements their significance in regions such as Asia and Africa, where goats play a crucial role in providing nutrition and income. Asia and Africa have seen substantial growth in DG numbers, highlighting the importance of promoting autochthonous breeds with dairy traits. These breeds are more resilient to climate change and produce milk with higher fat and protein content compared to most European breeds. To maximize these benefits, it is crucial to prioritize the research and conservation of these local breeds as a way to ensure ongoing production success and preserve genetic diversity in the species. On the other hand, the European approach toward reaching peak efficiency in goat milk production while also prioritizing animal welfare in environmentally friendly and ethically responsible settings will be crucial. Moreover, DGs play a crucial role in promoting sustainable agriculture using holistic and ecological farming practices. Clearly, there are many choices for action; utilizing a circular economy mindset can involve utilizing outputs such as manure from diverse DGPSs as organic fertilizer while implementing controlled grazing to help prevent forest fires. It also offers another way to reevaluate the ecosystem services of goats.

Animal well-being, coupled with improved reproductivity and productive efficiency, as well as the ethical use of natural resources in a sustainable manner, are expected to encourage a stronger dedication from all members of the goat milk production chain. This situation is projected to lead to increased innovation throughout the production chain because of the higher demands from a more informed and environmentally conscious society. In this regard, this strategy should evolve into a situation where all participants in the production process, including consumers, come together for mutual benefits under a win–win strategy. Fortunately, there is an increasing relevance in scientific studies regarding goat milk and its products, compared to its previous marginal status. Moreover, the increasing worldwide demand for goat milk and its products is expected to enhance the lives of producers, processors, and consumers worldwide. Interestingly, there are numerous significant global initiatives focused on scientific collaboration and sharing information regarding nutrition, reproductive, and genetic technologies related to the safety and nutraceutical quality of goat milk and its products while being contextualized in different DGPSs. Therefore, creating additional opportunities for researchers, producers, policymakers, and development workers to come together is fundamental to align interests and needs and exchange knowledge on effective goat farmer support strategies, environmental management, and consumer education. To effectively make a difference in reproductive and milk production efficiency, it is central to ensure that all these strategies are effectively aligned with the various DGPSs discussed in this study. The above should encourage increased production while prioritizing social and environmental responsibility. This should ideally create opportunities for growth within the goat industry, particularly for small producers and their families; so be it.

## Figures and Tables

**Table 1:** A summary of the main classifications of dairy goat production systems in the world.

Based on	Systems	Region/Country [Ref]
Level of production and use of natural resources	Traditional (meat milk production); Intensive milk production; Permanently indoors; Pasture with different grazing times; Confined; Grazing; Continuous grazing; Seasonal grazing; Transhumance; Use of natural and/or cultivated pastures; Pastoral; and Organic	Europe [23,24,34,35,77]
Characteristics of raw milk and water used in milking	Smallholder dairy farms: Conventional; Traditional; and Emerging	Brazilian semi-arid region [78]
Characteristics of farms and management practices	Cluster 1. Large, semi-intensive, high producing and investing farms; Cluster 2. Semi-extensive, low-input, traditional farms; Cluster 3. Medium-sized, semi-intensive, low replacement rate and less grazing farms; and Cluster 4. Semi-extensive, low-input, traditional farms on expansion, producing heavy weight kids’ carcasses	Greece [35]
Level of use of natural resources	Intensive; Semi-intensive; Extensive; Semi-extensive, Dual-purpose; Specialized; and Transhumant grazing-based	France, Greece, Italy, and Spain [12]
Type of resource used	Extensive; Tethering system with grazing; Confined; Semi-intensive; and Intensive	East and South Asian region [25]
	Pastoral; Pastoral range grazing; Mixed; Kid selling; Village; Migratory; and Intensive	Central and West Asia and North Africa region [25]
	Mixed; and low-input dairy goat	West African region [25]
	Extensive; Free ranging; and Pastoralist	East and Central Africa [25]
	Pastoral; Agro-pastoral; and Low/medium-input production	South African region [25]
	Extensive; Semi-extensive; Semi-intensive; Daytime grazing and nighttime confinement with supplementation; Nomadic system; Transhumance production; Sedentary production; Intensive; Indoor system; Flock system; and Traditional	Europe continent [25]
	Extensive or Traditional; Semi-extensive or Advanced; Intensive; Extensive system in rangelands; Extensive system where goats graze crop residues; and Intensive with use of grain and irrigated cut forages	American Continent [25]
Management practices	Backyard system (Extensive system); Smallholder goat production (Extensive system); Smallholder (Semi-intensive production system); Smallholder (Intensive production system); Medium-to-large flock on Extensive production system; Medium to large flock on Semi-intensive production system; and Large flock on Intensive production system	India [26]
Technical and economic indicators	Cluster 1. Grazing systems with high feed supply; Cluster 2. Indoor systems without associated crops; Cluster 3. Pastoral systems; and Cluster 4. Indoor systems with associated crops	Andalusia (Southern Spain) [36]
Analysis of typologies, standing out the importance of the variable farm size	Group I—Extensive systems with low input; Group II—Semi-intensive systems with high input; and Group III—Semi-intensive systems with moderate input	Northeast Brazil [27]
